# An overview of hand postures and aging on morphological changes of the median nerve

**DOI:** 10.1186/s40101-019-0201-6

**Published:** 2019-08-08

**Authors:** Ping Yeap Loh, Wen Liang Yeoh, Satoshi Muraki

**Affiliations:** 1 Department of Human Science, Faculty of Design, Kyushu University, Fukuoka, Japan; 2 Department of Human Science, Graduate School of Design, Kyushu University, Fukuoka, Japan

**Keywords:** Deformation, Median nerve cross-sectional area, Median nerve diameter

## Abstract

**Background:**

High-resolution ultrasound is being widely used in carpal tunnel examination to understand morphological and biomechanical characteristics of the median nerve and surrounding anatomy structures.

**Main body:**

Healthy young and elderly men were recruited. The median nerve at proximal wrist region was examined by ultrasound imaging technique. A total of seven wrist angle was examined. Generally, the median nerve cross-sectional area of the elderly group is significantly larger than the young group.

**Short conclusion:**

Wrist posture in greater flexion or extension caused a larger decrease in the median nerve cross-sectional area across both groups.

## Background

Carpal tunnel syndrome (CTS) is defined as compression neuropathy of the median nerve at the carpal tunnel level, and it is one of the most common reported peripheral nerve entrapment syndromes of the upper limb [1]. Multiple factors contribute to work-related CTS such as personal characteristics, biomechanical, psychosocial, and organizational factors. In recent years, several ultrasound studies investigated the impact of biomechanical stresses such as finger flexor tendon gliding and external compression on the deformation of the median nerve in healthy and CTS individuals [2–5]. However, evidence of the present research still remains inconclusive and is unable to address comprehensively the pathophysiology of CTS. Therefore, investigation of morphological changes and biomechanical relationship of the structures within the carpal tunnel is warranted for a wider understanding of median nerve deformation.

**Fig. 1:**
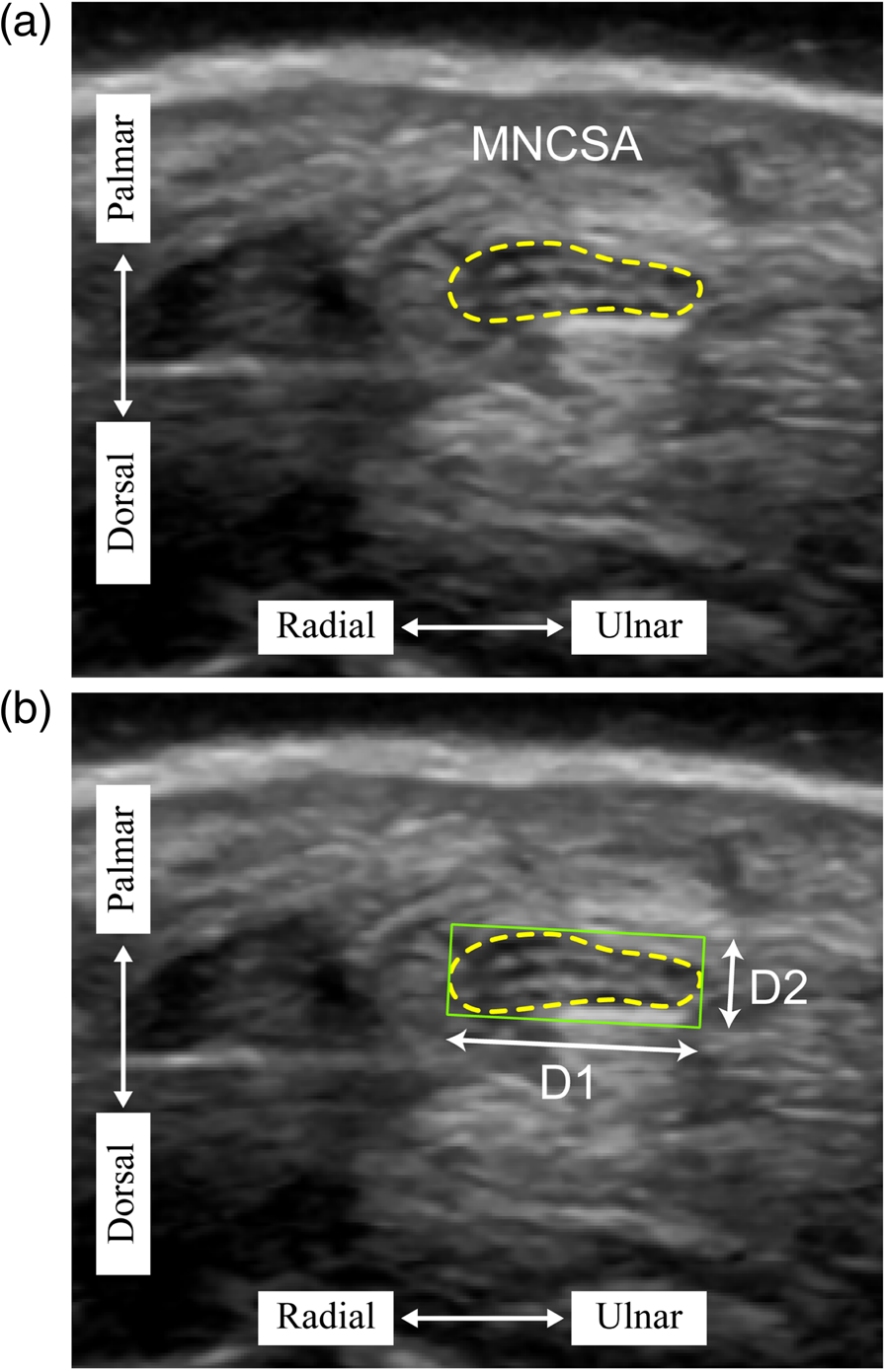
Quantification of (**a**) median nerve cross-sectional area by tracing method and (**b**) median nerve diameter by minimum bounding rectangle

The underlying biomechanical factors of the active wrist and finger movements causing changes in the median nerve can be examined and analyzed using high-resolution ultrasound imaging technique [6–8]. The common use morphological measurements are cross-sectional area by tracing method (Fig. 1a) and diameter (longitudinal diameter, D1; vertical diameter, D2) of the nerve by minimum bounding rectangle method (Fig. 1b). The main objective is to extend the confirmatory study to our previous findings [9, 10] on the median nerve changes by comparing the differences of median nerve cross-sectional area (MNCSA) between young and elderly participants at different wrist angle.

## Methods

This study was approved by the Ethics Committee of the Faculty of Design at Kyushu University. All participants provided a written informed consent. Participants were divided into young (*n* = 21, 24.9 ± 2.8 years) and elderly (*n* = 31, 70.9 ± 5.2 years) groups. The inclusion criteria are right-hand dominant by Edinburg Handedness Inventory [11], no previous CTS and wrist injury history by self-report, and the MNCSA measurement of both hands fall within mean ± 2SD. The eligible data for statistical analysis is summarized in Table 1.

**Table 1:** Eligible data after inclusion criteria

Group (years) [9, 10]	Age range (years)	Eligible wrists	
		Right	Left
Young (24.9 ± 2.8)		21	21
Elderly (70.9 ± 5.2)	61.8 – 80.2	31	30

Ultrasound images were obtained via the LOGIQ e ultrasound system (GE Healthcare, Milwaukee, WI, USA) with a 12L-RS transducer (imaging frequency bandwidth of 5–13 MHz). The ultrasound protocol included wrist examination at 7 different passive wrist angle (neutral (0°); 15°, 30°, and 45° extension; and 15°, 30°, and 45° flexion) and the MNCSA was quantified using ImageJ (National Institutes of Health) [9, 10]. Our previous study indicated that active and passive wrist holding position did not cause significant differences in the measured MNCSA [12]. The median nerve was identified by the hyperechogenic rim and MNCSA was quantified by tracing along the hyperechognic rim (Fig. 1a) and median nerve diameter by minimum bounding rectangle method (Fig. 1b).

Statistical analysis was performed using SPSS version 21.0 software (IBM Corporation, Chicago, IL, USA). Two-way analysis of variance was conducted with wrist angle and age group as factors to examine differences in MNCSA, median nerve diameter (D1 and D2) for both right and left wrist. The significance level was set at < 0.05 (5%). All data are presented in mean ± SD.

## Results

### MNCSA

**Fig. 2:**
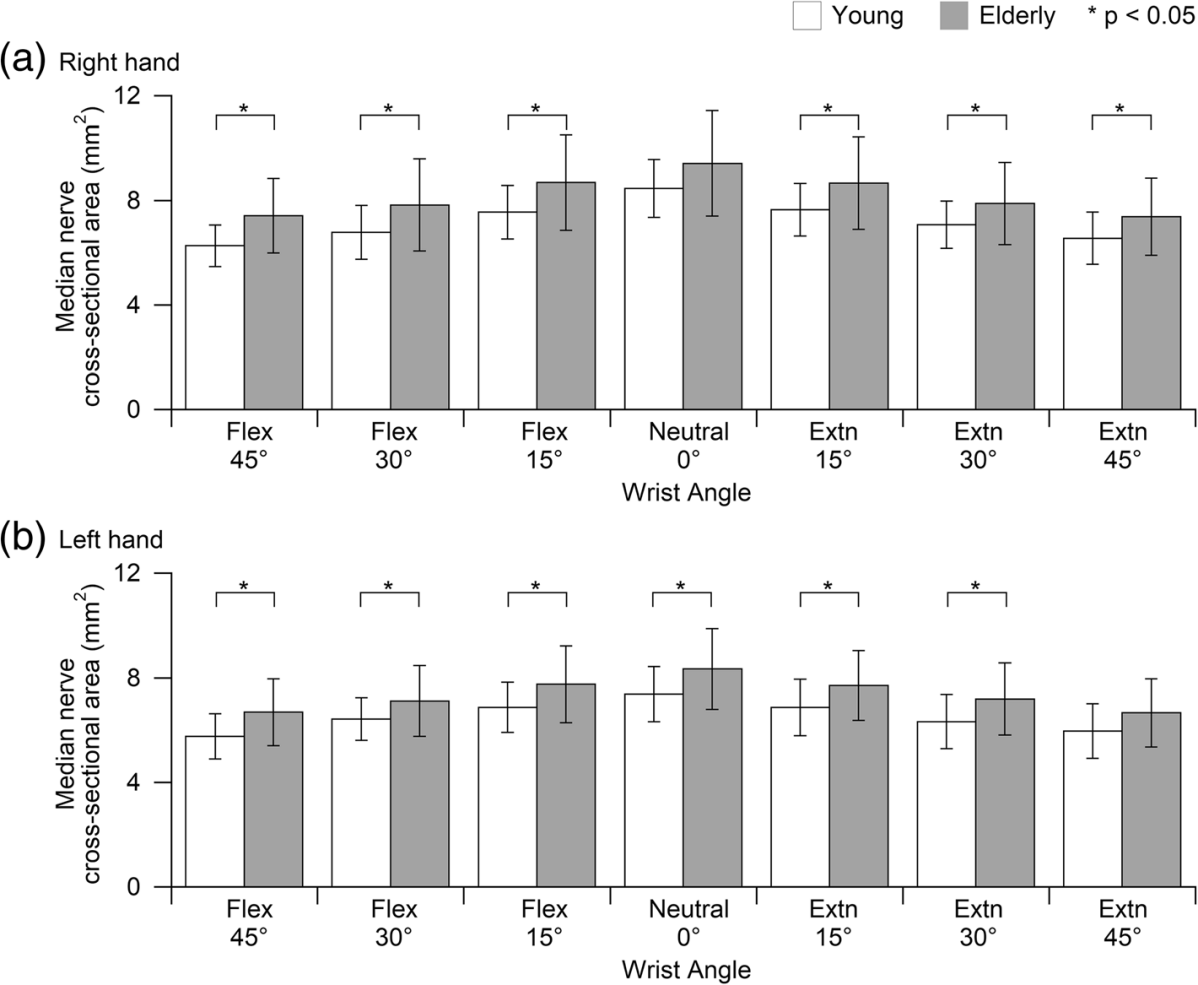
Comparison of median nerve cross-sectional area (MNCSA, mm^2^) between young and elderly men at different wrist angles (**a**) right hand and (**b**) left hand

Wrist angle and age group factors cause significantly difference (*p* < 0.05) between the young and elderly group at both hands. No significant interaction was found between wrist angle and age group. Wrist angle deviates from neutral lead to a decrease in median nerve cross-sectional area (MNCSA). Overall, the elderly have a larger MNCSA compared to young participants at different wrist angle (Fig. 2).

### Median nerve diameter (D1 and D2)

**Fig. 3:**
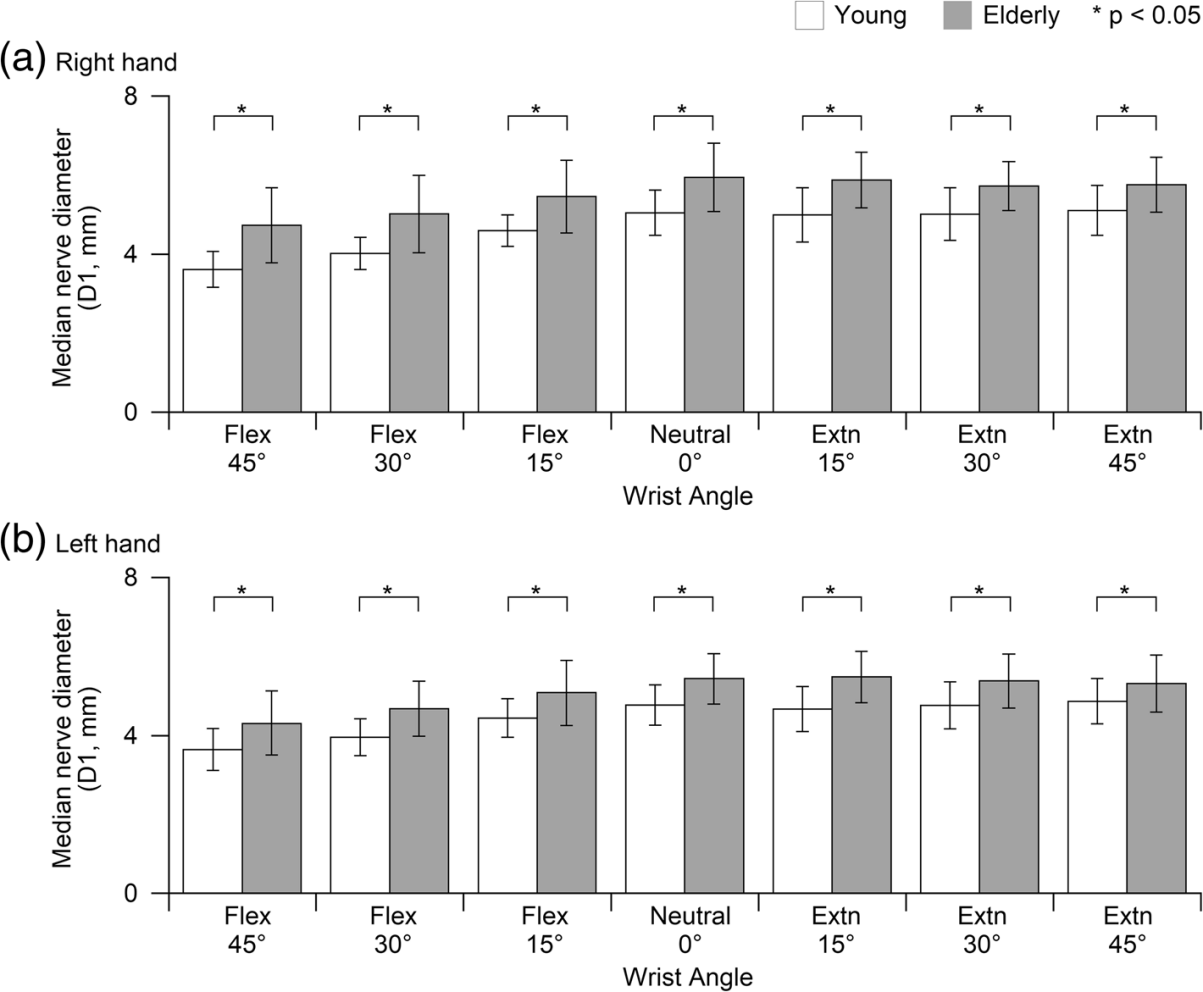
Comparison of median nerve diameter, D1 (mm) between young and elderly men at different wrist angles (**a**) right hand and (**b**) left hand

**Fig. 4:**
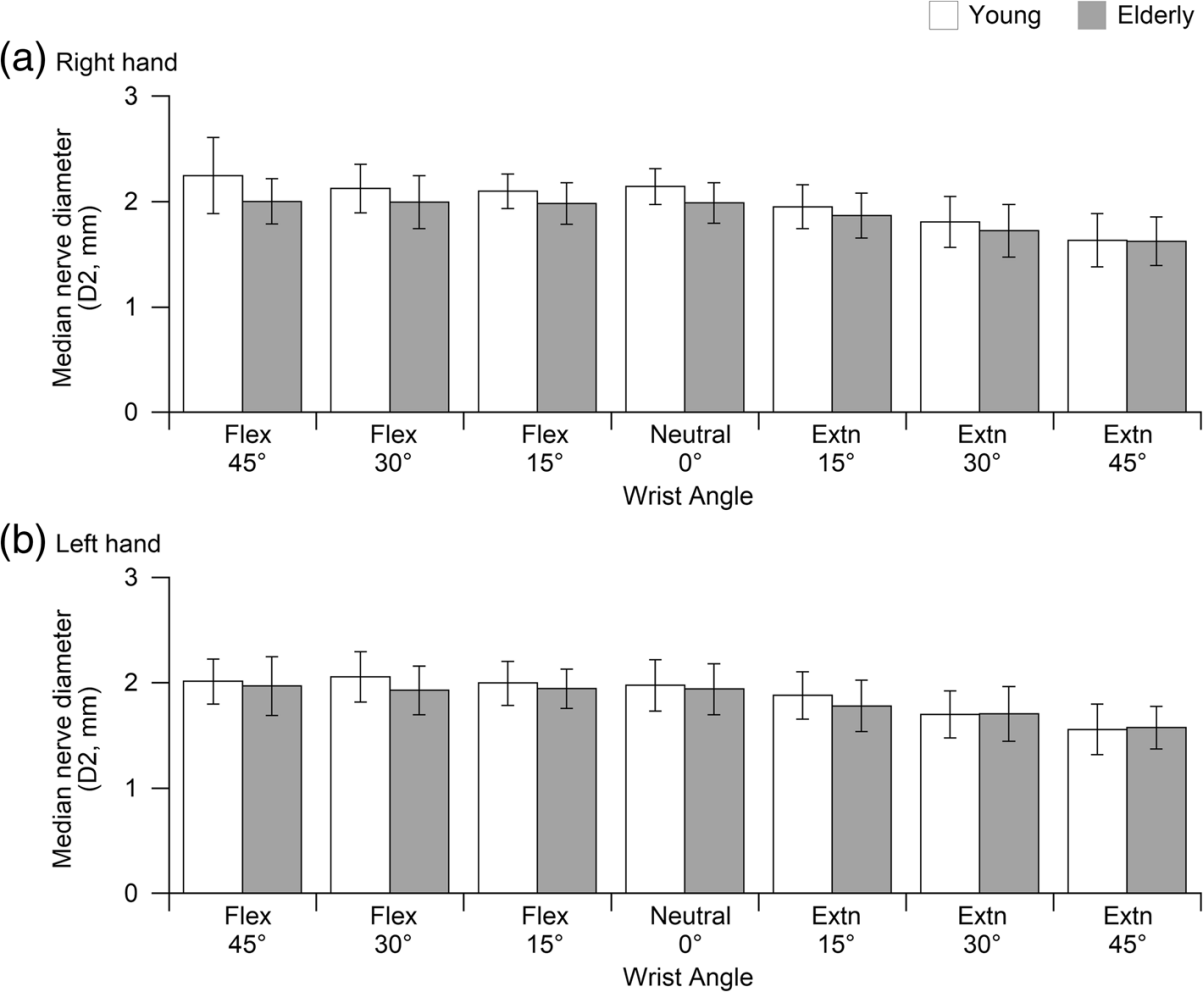
Comparison of median nerve diameter, D2 (mm) between young and elderly men at different wrist angles (**a**) right hand and (**b**) left hand

For D1, wrist angle and age group factor cause significantly difference (*p* < 0.05) between the young and elderly group at both hands (Fig. 3a, b). For D2, only wrist angle factor causes significantly difference (*p* < 0.05) between the young and elderly group at both hands (Fig. 4a, b). Similar to MNCSA, no significant interaction was found between wrist angle and age group.

## Discussion

The comparison results between young and elderly suggested that wrist flexion-extension is the main factor leading to significant changes in the MNCSA as described by previous studies [13]. Subsequently, the ultrasound examination was conducted with passive wrist holding posture as previous study [12]. Additionally, a maximal wrist radial-ulnar deviation in a neutral wrist position, 30° flexion, and 30° extension did not cause further deformation of the median nerve when compared to a neutral, non-radial/ulnar deviated posture [13].

The aging process has led to changes in the morphological characteristics such as myelin and nerve fibers, and peripheral nerve functions such as the reduction in velocity [14–16]. However, peripheral nerve cross-sectional area reference values, such as median nerve, at different age group still remain debatable [17, 18]. Our findings indicate larger MNCSA and D1 among elderly men in comparison to young men (Figs. 3 and 4) at different wrist posture. This could due to the deterioration of the peripheral nerve among elderly such as increased thickness of myelin sheath [19].

Subsequently, a detailed examination of the relationship between the wrist posture and deformation of the median nerve was conducted by considering the functional range of motion of the wrist joint in daily work tasks. The MNCSA and the diameter of the median nerve (D1 and D2) in the wrist in different postures including 15°, 30°, and 45° of flexion and extension were analyzed and compared to the neutral wrist (0°) as a control [9, 10]. In general, our findings are in line with previous studies. Wrist posture in flexed or extended position caused a significant decrease in the MNCSA. Additionally, wrist flexion and extension caused a significant decrease in D1 and D2 respectively (Figs. 3 and 4). Notably, a greater flexion or extension angle resulted in greater deformation of the MNCSA and median nerve diameter. Based on findings, measurements of the median nerve in the dominant hand are larger than those in the non-dominant hand and measurements among young men are larger than among young women [9]. On the other hand, trends of median nerve deformation are similar in young and elderly men regardless of larger measurements among elderly men (Figs. 2, 3, and 4). In summary, wrist posture changes impose biomechanical stress on the carpal tunnel and affect the behavior of the median nerve.

Various joint angles of the metacarpophalangeal joint, proximal interphalangeal joint, and distal interphalangeal joint in each finger posture cause a change in the MNCSA and median nerve diameter. Additionally, force exertion such as power gripping causes a further reduction of the MNCSA [20]. In conjunction with active finger movements, wrist posture exists as an important influential factor of deformation of the median nerve. For instance, deformation of the median nerve during wrist flexion or extension in each finger posture is higher than that in a neutral wrist. Observed results may help to identify finger movements that contribute to work-related CTS. These findings provide broader perspectives regarding biomechanical stress from active finger tendon gliding on the median nerve.

The application of ultrasound imaging on median nerve morphological study is useful for the aging workforce. For instance, there is an increase using of computer use in daily work that may contribute to work-related musculoskeletal disorders such as CTS. However, it remains debatable whether repetitiveness and duration of computer work tasks are risk factors of work-related CTS. Morphological investigation of the median nerve among young participants revealed that a 30-min keyboard typing task can have an acute impact on the median nerve [21]. Subsequently, the wrist kinematics when typing using keyboard of higher tilted slopes results in a greater wrist extension, which could possibly link to higher changes of the median nerve. Therefore, changes of median nerve morphological parameter such as MNCSA and diameter may serve as an indicator to understand the impact of work task on the wrist.

## Summary

This paper presents the differences between young and elderly group and summarizes the evidence related to morphologic adaptability of the median nerve using a well-designed experimental protocol and physical task exposure. However, there are still unanswered questions as well as unidentified relationships between different physical work exposures and the pathophysiology of CTS. Additionally, translation of the findings from this study to ergonomics and clinical practices is important for CTS prevention. A longitudinal study of different occupations is needed to identify risk factors and develop an early-preventive intervention plan for CTS.

## Data Availability

The dataset analyzed for the current study is available from the corresponding author on reasonable request.

## Abbreviations

CTS: Carpal tunnel syndrome
D1: Longitudinal diameter
D2: Vertical diameter
MNCSA: Median nerve cross-sectional area
